# Supplementary material for: Pyruvate Dehydrogenase Kinase 1 inhibition mediated oxidative phosphorylation enhancement in cartilage promotes osteoarthritis progression
Source: BMC Musculoskelet Disord. 2023 Jul 20;24:597. doi: 10.1186/s12891-023-06585-6 (PMC10357736; doi:10.1186/s12891-023-06585-6)
Supplement: Supplementary file 1 — Additional file 1. [file 12891_2023_6585_MOESM1_ESM.zip › MF.Gsea.1653627316242/GOMF_DAMAGED_DNA_BINDING.html]

Details for gene set GOMF\_DAMAGED\_DNA\_BINDING[GSEA]

|  || Dataset | 55457\_collapsed\_to\_symbols.cls.cls #OA\_versus\_normal.cls.cls #OA\_versus\_normal\_repos |
| Phenotype | cls.cls#OA\_versus\_normal\_repos |
| Upregulated in class | OA |
| GeneSet | GOMF\_DAMAGED\_DNA\_BINDING |
| Enrichment Score (ES) | 0.35809958 |
| Normalized Enrichment Score (NES) | 1.5224354 |
| Nominal p-value | 0.010178117 |
| FDR q-value | 0.18650815 |
| FWER p-Value | 1.0 |
Table: GSEA Results Summary

  

Fig 1: Enrichment plot: GOMF\_DAMAGED\_DNA\_BINDING      
 Profile of the Running ES Score & Positions of GeneSet Members on the Rank Ordered List

  

| SYMBOL | TITLE | RANK IN GENE LIST | RANK METRIC SCORE | RUNNING ES | CORE ENRICHMENT || 1 | RPA3 | replication protein A3 [Source:HGNC Symbol;Acc:HGNC:10291] | 13 | 1.159 | 0.0526 | Yes |
| 2 | POLB | DNA polymerase beta [Source:HGNC Symbol;Acc:HGNC:9174] | 93 | 0.923 | 0.0892 | Yes |
| 3 | MPG | N-methylpurine DNA glycosylase [Source:HGNC Symbol;Acc:HGNC:7211] | 277 | 0.768 | 0.1104 | Yes |
| 4 | RPA2 | replication protein A2 [Source:HGNC Symbol;Acc:HGNC:10290] | 339 | 0.727 | 0.1393 | Yes |
| 5 | OGG1 | 8-oxoguanine DNA glycosylase [Source:HGNC Symbol;Acc:HGNC:8125] | 453 | 0.681 | 0.1621 | Yes |
| 6 | RPA1 | replication protein A1 [Source:HGNC Symbol;Acc:HGNC:10289] | 571 | 0.632 | 0.1822 | Yes |
| 7 | MSH2 | mutS homolog 2 [Source:HGNC Symbol;Acc:HGNC:7325] | 739 | 0.584 | 0.1962 | Yes |
| 8 | POLD1 | "DNA polymerase delta 1, catalytic subunit [Source:HGNC Symbol;Acc:HGNC:9175]" | 811 | 0.566 | 0.2169 | Yes |
| 9 | RAD1 | RAD1 checkpoint DNA exonuclease [Source:HGNC Symbol;Acc:HGNC:9806] | 987 | 0.521 | 0.2273 | Yes |
| 10 | XRCC5 | X-ray repair cross complementing 5 [Source:HGNC Symbol;Acc:HGNC:12833] | 1018 | 0.513 | 0.2487 | Yes |
| 11 | DCLRE1A | DNA cross-link repair 1A [Source:HGNC Symbol;Acc:HGNC:17660] | 1073 | 0.500 | 0.2677 | Yes |
| 12 | KDM4D | lysine demethylase 4D [Source:HGNC Symbol;Acc:HGNC:25498] | 1180 | 0.480 | 0.2817 | Yes |
| 13 | POT1 | protection of telomeres 1 [Source:HGNC Symbol;Acc:HGNC:17284] | 1340 | 0.451 | 0.2901 | Yes |
| 14 | DCLRE1B | DNA cross-link repair 1B [Source:HGNC Symbol;Acc:HGNC:17641] | 1521 | 0.426 | 0.2958 | Yes |
| 15 | PCNA | proliferating cell nuclear antigen [Source:HGNC Symbol;Acc:HGNC:8729] | 1557 | 0.419 | 0.3125 | Yes |
| 16 | XPA | "XPA, DNA damage recognition and repair factor [Source:HGNC Symbol;Acc:HGNC:12814]" | 1615 | 0.411 | 0.3271 | Yes |
| 17 | PNKP | polynucleotide kinase 3'-phosphatase [Source:HGNC Symbol;Acc:HGNC:9154] | 1662 | 0.404 | 0.3422 | Yes |
| 18 | ERCC4 | "ERCC excision repair 4, endonuclease catalytic subunit [Source:HGNC Symbol;Acc:HGNC:3436]" | 1696 | 0.400 | 0.3581 | Yes |
| 19 | XRCC1 | X-ray repair cross complementing 1 [Source:HGNC Symbol;Acc:HGNC:12828] | 2182 | 0.340 | 0.3361 | No |
| 20 | DDB1 | damage specific DNA binding protein 1 [Source:HGNC Symbol;Acc:HGNC:2717] | 2302 | 0.326 | 0.3419 | No |
| 21 | APTX | aprataxin [Source:HGNC Symbol;Acc:HGNC:15984] | 2702 | 0.279 | 0.3237 | No |
| 22 | ERCC1 | "ERCC excision repair 1, endonuclease non-catalytic subunit [Source:HGNC Symbol;Acc:HGNC:3433]" | 2948 | 0.255 | 0.3165 | No |
| 23 | NTHL1 | nth like DNA glycosylase 1 [Source:HGNC Symbol;Acc:HGNC:8028] | 3219 | 0.228 | 0.3060 | No |
| 24 | DDB2 | damage specific DNA binding protein 2 [Source:HGNC Symbol;Acc:HGNC:2718] | 3257 | 0.225 | 0.3135 | No |
| 25 | XPC | "XPC complex subunit, DNA damage recognition and repair factor [Source:HGNC Symbol;Acc:HGNC:12816]" | 3278 | 0.222 | 0.3223 | No |
| 26 | ERCC2 | "ERCC excision repair 2, TFIIH core complex helicase subunit [Source:HGNC Symbol;Acc:HGNC:3434]" | 3544 | 0.198 | 0.3108 | No |
| 27 | RBBP8 | "RB binding protein 8, endonuclease [Source:HGNC Symbol;Acc:HGNC:9891]" | 3739 | 0.182 | 0.3041 | No |
| 28 | CUL4B | cullin 4B [Source:HGNC Symbol;Acc:HGNC:2555] | 3783 | 0.179 | 0.3090 | No |
| 29 | NEIL3 | nei like DNA glycosylase 3 [Source:HGNC Symbol;Acc:HGNC:24573] | 4006 | 0.156 | 0.2989 | No |
| 30 | NBN | nibrin [Source:HGNC Symbol;Acc:HGNC:7652] | 4136 | 0.143 | 0.2955 | No |
| 31 | POLQ | DNA polymerase theta [Source:HGNC Symbol;Acc:HGNC:9186] | 4293 | 0.129 | 0.2893 | No |
| 32 | ERCC5 | "ERCC excision repair 5, endonuclease [Source:HGNC Symbol;Acc:HGNC:3437]" | 4380 | 0.122 | 0.2883 | No |
| 33 | XRCC6 | X-ray repair cross complementing 6 [Source:HGNC Symbol;Acc:HGNC:4055] | 4699 | 0.096 | 0.2679 | No |
| 34 | AUNIP | aurora kinase A and ninein interacting protein [Source:HGNC Symbol;Acc:HGNC:28363] | 4802 | 0.087 | 0.2640 | No |
| 35 | BLM | BLM RecQ like helicase [Source:HGNC Symbol;Acc:HGNC:1058] | 4843 | 0.084 | 0.2648 | No |
| 36 | TP53BP1 | tumor protein p53 binding protein 1 [Source:HGNC Symbol;Acc:HGNC:11999] | 4946 | 0.076 | 0.2604 | No |
| 37 | POLH | DNA polymerase eta [Source:HGNC Symbol;Acc:HGNC:9181] | 4958 | 0.076 | 0.2630 | No |
| 38 | FEN1 | flap structure-specific endonuclease 1 [Source:HGNC Symbol;Acc:HGNC:3650] | 5212 | 0.056 | 0.2459 | No |
| 39 | MUTYH | mutY DNA glycosylase [Source:HGNC Symbol;Acc:HGNC:7527] | 5252 | 0.052 | 0.2452 | No |
| 40 | HMGB2 | high mobility group box 2 [Source:HGNC Symbol;Acc:HGNC:5000] | 5272 | 0.050 | 0.2461 | No |
| 41 | FANCG | FA complementation group G [Source:HGNC Symbol;Acc:HGNC:3588] | 5675 | 0.017 | 0.2156 | No |
| 42 | UNG | uracil DNA glycosylase [Source:HGNC Symbol;Acc:HGNC:12572] | 6078 | -0.011 | 0.1847 | No |
| 43 | TP63 | tumor protein p63 [Source:HGNC Symbol;Acc:HGNC:15979] | 6274 | -0.025 | 0.1707 | No |
| 44 | POLI | DNA polymerase iota [Source:HGNC Symbol;Acc:HGNC:9182] | 7511 | -0.119 | 0.0800 | No |
| 45 | SMC6 | structural maintenance of chromosomes 6 [Source:HGNC Symbol;Acc:HGNC:20466] | 8043 | -0.158 | 0.0459 | No |
| 46 | BRCA1 | BRCA1 DNA repair associated [Source:HGNC Symbol;Acc:HGNC:1100] | 8417 | -0.187 | 0.0255 | No |
| 47 | CRY2 | cryptochrome circadian regulator 2 [Source:HGNC Symbol;Acc:HGNC:2385] | 8536 | -0.196 | 0.0254 | No |
| 48 | ERCC3 | "ERCC excision repair 3, TFIIH core complex helicase subunit [Source:HGNC Symbol;Acc:HGNC:3435]" | 8669 | -0.206 | 0.0246 | No |
| 49 | APEX1 | apurinic/apyrimidinic endodeoxyribonuclease 1 [Source:HGNC Symbol;Acc:HGNC:587] | 8767 | -0.213 | 0.0270 | No |
| 50 | RAD23B | "RAD23 homolog B, nucleotide excision repair protein [Source:HGNC Symbol;Acc:HGNC:9813]" | 8777 | -0.215 | 0.0362 | No |
| 51 | MSH6 | mutS homolog 6 [Source:HGNC Symbol;Acc:HGNC:7329] | 8846 | -0.221 | 0.0411 | No |
| 52 | H2AX | H2A.X variant histone [Source:HGNC Symbol;Acc:HGNC:4739] | 8979 | -0.230 | 0.0415 | No |
| 53 | RAD23A | "RAD23 homolog A, nucleotide excision repair protein [Source:HGNC Symbol;Acc:HGNC:9812]" | 9028 | -0.234 | 0.0486 | No |
| 54 | MSH3 | mutS homolog 3 [Source:HGNC Symbol;Acc:HGNC:7326] | 9796 | -0.301 | 0.0028 | No |
| 55 | WRN | WRN RecQ like helicase [Source:HGNC Symbol;Acc:HGNC:12791] | 10010 | -0.323 | 0.0011 | No |
| 56 | REV1 | REV1 DNA directed polymerase [Source:HGNC Symbol;Acc:HGNC:14060] | 10500 | -0.373 | -0.0198 | No |
| 57 | TDG | thymine DNA glycosylase [Source:HGNC Symbol;Acc:HGNC:11700] | 10728 | -0.396 | -0.0191 | No |
| 58 | CREBBP | CREB binding protein [Source:HGNC Symbol;Acc:HGNC:2348] | 10881 | -0.414 | -0.0118 | No |
| 59 | NEIL1 | nei like DNA glycosylase 1 [Source:HGNC Symbol;Acc:HGNC:18448] | 10963 | -0.425 | 0.0016 | No |
| 60 | RPS3 | ribosomal protein S3 [Source:HGNC Symbol;Acc:HGNC:10420] | 11245 | -0.463 | 0.0011 | No |
| 61 | EP300 | E1A binding protein p300 [Source:HGNC Symbol;Acc:HGNC:3373] | 11741 | -0.540 | -0.0125 | No |
| 62 | RECQL4 | RecQ like helicase 4 [Source:HGNC Symbol;Acc:HGNC:9949] | 11927 | -0.578 | -0.0001 | No |
| 63 | HMGB1 | high mobility group box 1 [Source:HGNC Symbol;Acc:HGNC:4983] | 12170 | -0.636 | 0.0104 | No |
| 64 | DCLRE1C | DNA cross-link repair 1C [Source:HGNC Symbol;Acc:HGNC:17642] | 12818 | -1.002 | 0.0064 | No |
Table: GSEA details [plain text format]

  

Fig 2: GOMF\_DAMAGED\_DNA\_BINDING      
 Blue-Pink O' Gram in the Space of the Analyzed GeneSet

  

Fig 3: GOMF\_DAMAGED\_DNA\_BINDING: Random ES distribution      
 Gene set null distribution of ES for **GOMF\_DAMAGED\_DNA\_BINDING**
